# Transcriptome signature in the blood of neuromyelitis optica spectrum disorder under steroid tapering

**DOI:** 10.3389/fimmu.2025.1508977

**Published:** 2025-02-03

**Authors:** Ryohei Yamamura, Makoto Kinoshita, Yoshiaki Yasumizu, Tomohiro Yata, Keigo Kihara, Daisuke Motooka, Naoyuki Shiraishi, Yasuko Sugiyama, Shohei Beppu, Hisashi Murata, Naoshi Koizumi, Itsuki Sano, Toru Koda, Tatsusada Okuno, Hideki Mochizuki

**Affiliations:** ^1^ Department of Neurology, Graduate School of Medicine, Osaka University, Suita, Osaka, Japan; ^2^ Department of Experimental Immunology, Immunology Frontier Research Center, Osaka University, Suita, Osaka, Japan; ^3^ Integrated Frontier Research for Medical Science Division, Institute for Open and Transdisciplinary Research Initiatives (OTRI), Osaka University, Suita, Osaka, Japan; ^4^ Genome Information Research Center, Research Institute for Microbial Diseases, Osaka University, Suita, Osaka, Japan

**Keywords:** NMOSD, steroid, transcriptome signature, IL-10, interferon

## Abstract

**Background:**

The advent of biologics has significantly transformed treatment strategies for neuromyelitis optica spectrum disorder (NMOSD). However, there are no biomarkers that predict relapses associated with steroid tapering; therefore, it is critical to identify potential indicators of disease activity. In this study, we collected peripheral blood mononuclear cells (PBMCs) from NMOSD patients during steroid tapering and performed bulk RNA sequencing to analyze changes in immune dynamics caused by steroid reduction.

**Methods:**

PBMCs were collected at 3–5 timepoints from 10 NMOSD patients at our hospital (including one relapse case), and bulk RNA sequencing was performed. All patients were positive for anti-AQP4 antibodies and had no history of biologic use.

**Results:**

In one relapsed patient, gene groups with decreased expression at relapse were observed predominantly in monocytes, with upregulation in anti-inflammatory pathways such as IL-10, while the upregulated genes were related to interferon signaling. Moreover, after steroid tapering, in non-relapsed patients, genes with increased expression were enriched in inflammatory pathways, represented by interferon signaling, while genes with decreased expression were enriched in pathways related to IL-10 and glucocorticoid receptors. Weighted gene co-expression network analysis identified modules that correlated with steroid dosage, and the modules inversely correlated with steroid dosage were enriched in monocytes, with marked immune signature of interferon pathway.

**Conclusion:**

This study identified peripheral blood transcriptome signatures that could lead to the identification of clinically relevant NMOSD disease activity biomarkers, and further highlights the pivotal role of interferon and IL-10 signaling in NMOSD.

## 1 Introduction

With the advent of various monoclonal antibodies targeting molecules involved in the pathogenesis of neuromyelitis optica spectrum disorder (NMOSD) (1, 2), there has been marked progress in the prevention therapy strategy of the patients. NMOSD is an autoimmune inflammatory disease of the central nervous system. The presence of the highly disease-specific anti–aquaporin 4 (anti-AQP4) autoantibody is the hallmark of the disease (3), and patients with NMOSD experience severe attacks that predominantly involve the optic nerves, spinal cord, and brainstem (4). Accumulating pathological and experimental evidence has shown that anti-AQP4 antibodies induce astrocytic necrosis after binding to astrocytes that highly express AQP4 (5). These antibodies subsequently activate the classical complement pathway and are deposited as an immune complex, resulting in a unique form of the “rim and rosette distribution pattern” (6). In addition, levels of IL-6, a major inflammatory cytokine, are elevated in the blood and cerebrospinal fluid of NMOSD patients (7). IL-6 has been reported to stimulate plasmablast survival, AQP4-IgG secretion, and loss of blood–brain barrier integrity, and also to induce the differentiation and activation of inflammatory T cells (7). Finally, in addition to the plasmablast population, various B cell subsets expressing CD19 or CD20 are known to play major roles in producing anti-AQP4 antibodies (8).

Despite outstanding results in randomized clinical trials of eculizumab (9), satralizumab (10), inebilizumab (11), and rituximab (12), markers of specific disease activity are necessary to achieve monotherapy without concomitant steroid administration. This is crucial because in real-world clinical settings, patients may experience mild to moderate relapses during steroid tapering after the initiation of certain monoclonal antibodies (13). Additionally, in several Asian countries, steroids and immunosuppressants are still widely used for financial reasons instead of monoclonal antibodies, further highlighting the need to identify markers that reflect the disease activity of NMOSD (14).

It has been shown that patients with NMOSD undergo frequent relapses without prevention therapy (15, 16). Previous literature shows inconsistent correlation of anti-AQP4 antibody titers and disease activity (17). IL-6 is shown to be the elevated cytokine in relapsing NMOSD compared to remission (18), and the number of CD27^high^CD38^high^CD180^−^ B cells is also reported to increase at the relapse phase of the disease (19).

In this study, we characterized the blood transcriptome signatures of NMOSD disease activity during steroid tapering by analyzing the gene signatures of peripheral blood mononuclear cells (PBMCs) at each steroid dosage. Bulk RNA sequencing (RNA-seq) was used to examine the immune dynamics in NMOSD patients. In addition, bulk RNA-seq was performed both during steroid tapering and in a case of clinical relapse to clarify gene changes specific to each condition. We expect that our observations will elucidate the disease activity of NMOSD and support clinical decisions related to safe steroid reducing regimen.

## 2 Materials and methods

### 2.1 Subjects and PBMC sample collection

PBMCs were collected from 10 patients with NMOSD at Osaka University Hospital who were diagnosed according to the 2015 NMOSD criteria (20). The protocol of this study involving human participants was reviewed and approved by the Ethics Committee of Osaka University (11298–36). All patients were positive for anti-AQP4 antibodies. Immunosuppressive drugs other than steroids were limited to azathioprine or the calcineurin inhibitor tacrolimus. Patients using biologics were excluded. The blood samples from a relapsed patient included in this study were taken at the timepoints of 570 days and 506 days before the relapse in the remission phase, and 50 days and 79 days after the relapse in the acute phase respectively. For the control samples, PBMCs derived from three patients positive for anti-acetylcholine receptor antibody, the hallmark of another autoantibody-mediated disease coined as myasthenia gravis, were collected. None of the control patients were taking steroids at the time of sample collection. Detailed information on the cohort is depicted in **Table 1** for patients with NMOSD, and in **Table 2** for the control samples. PBMCs were isolated with Ficoll-Paque™ PLUS (Cytiva Sweden AB, Uppsala, Sweden), then stored in a freezer at −80°C.

**Table 1:** Patient information with NMOSD.

Patient No.	Age at sample collection	Sex	Steroid dosage of each sample (mg/day)	Concomitant immunosuppressive drugs
1	51	Female	15	None
	51		10	azathioprine 75mg/day
	52		9	azathioprine 75mg/day
	52		5	azathioprine 75mg/day
	53		2.5	azathioprine 75mg/day
2	67	Female	10	None
	67		10	None
	67		15	None
	67		8	None
3	30	Female	6	None
	30		6	None
	30		6	None
	30		6	None
	31		10	None
	34		7.5	None
4	43	Female	14	None
	43		14	tacrolimus 2mg/day
	43		11	tacrolimus 3mg/day
	44		9	tacrolimus 3mg/day
	44		5	tacrolimus 3mg/day
	45		2	tacrolimus 3mg/day
5	51	Female	12.5	tacrolimus 3mg/day
	51		10	tacrolimus 3mg/day
	51		10	tacrolimus 3mg/day
	53		4	tacrolimus 3mg/day
6	46	Male	15	None
	46		14	None
	47		13	None
	47		10	None
7	41	Female	15	None
	43		10	tacrolimus 3mg/day
	43		6	tacrolimus 3mg/day
	46		3	tacrolimus 2.5mg/day
	49		2.5	tacrolimus 2.5mg/day
	49		1	tacrolimus 2.5mg/day
8	59	Female	6	tacrolimus 3mg/day
	61		3	tacrolimus 3mg/day
	63		2.5	tacrolimus 3mg/day
9	43	Female	15	None
	43		15	azathioprine(50mg/day and 25mg/day alternately every other day)
	45		5	azathioprine(50mg/day and 25mg/day alternately every other day)
	46		3	azathioprine 25mg/day
	48		3	azathioprine 25mg/day(every other day)
10	58	Female	5	tacrolimus 3mg/day
	58		10	tacrolimus 3mg/day
	59		5 (relapse)	tacrolimus 3mg/day
	59		5 (after acute-phase treatment)	tacrolimus 3mg/day

**Table 2:** Patient information for control samples.

Patient No.	Age at sample collection	Sex	Anti-acetylcholine receptor antibody
Control_1	57	Female	Positive
Control_2	49	Female	Positive
Control_3	74	Female	Positive

### 2.2 Bulk RNA-seq

Total RNA was extracted using the RNeasy Micro Kit (Qiagen, Valencia, CA, USA). Full-length cDNA was generated using a SMART-Seq HT Kit (Takara Bio, Kusatsu, Japan). An Illumina library was prepared using a NexteraXT DNA Library Preparation Kit (Illumina, San Diego, CA, USA) according to SMARTer kit instructions. Sequencing was performed on an Illumina NovaSeq 6000 sequencer (Illumina, San Diego, CA, USA) in the 100-base single-end mode. After adapter trimming using Trimmomatic, sequenced reads were mapped to the human reference genome sequence hg19 using TopHat version 2.1.1. The fragments per kilobase of exons per million mapped fragments (FPKM) value was calculated using Cuffnorm version 2.2.1. The raw data have been deposited in the NCBI’s Gene Expression Omnibus database (GEO GSE271586).

### 2.3 Principal component analysis and K-means analysis of bulk RNA-seq

Principal component analysis (PCA) and K-means analysis of bulk RNA-seq data were performed using iDEP (an integrated web application for differential expression and pathway analysis of RNA-seq data) (21). For the analysis, iDEP v2.0 was used, and parameters were set at the defaults. We used the top 2000 genes and set the number of clusters to four.

### 2.4 Pathway analysis

For pathway analysis, we used the Enricher application, and mapped out the pathways for each cluster. Either “Reactome 2022” or “WikiPathway 2023 Human” was used to calculate the data.

### 2.5 Weighted gene co-expression network analysis

A transformed matrix from normalized counts was used for WGCNA (v1.72.5) analysis using R 4.3.2 to identify significant modules that correlated with steroid dosage. Then, we calculated the adjacency using the adjacency function with power = 16. The adjacency matrix was then converted into a Topological Overlap Matrix (TOM) using TOMsimilarity, and a gene tree was calculated using hierarchical clustering against 1 - TOM with the “average” method. A dynamic tree cut was conducted with the following parameters; deepSplit = 0, pamRespectsDendro = FALSE, minModuleSize = 30. The eigengenes of each module were calculated using the moduleEigengenes function, and modules with strongly correlated eigengenes were merged, with MEDissThres set to 0.2. These eigengenes were used for the correlation with clinical information indicating the steroid dosage associated with each sample.

### 2.6 Differentially expressed gene analysis

To detect DEGs between relapse and remission samples, we used the edgeR package (v4.0.15) in R. We set the thresholds for significant differential expression at log_2_ fold change > 1 and a false discovery rate < 0.01.

### 2.7 Time course–based analysis of each cluster by K-means

Using the four clusters identified by K-means in Patient 10, we created a chronological landscape of gene expression levels for each patient other than Patient 10, on the basis of cluster 2 (downregulated during relapse) and cluster 3 (upregulated during relapse). For each patient, we converted the FPKM of each gene included in each cluster into base-2 logarithms. Using these values, we calculated the relative expression change value, which was determined by subtracting the mean log value across samples from the original log value. On the basis of the relative expression change value, we calculated the mean and standard deviation (SD) for each sample.

### 2.8 Gene set scoring in the single-cell RNA-seq dataset

To analyze the gene expression profile of each cluster, we used the Seurat package (v5.0.2) (22). A multimodal dataset of PBMCs, which was derived from single-cell RNA sequencing (scRNA-seq) combined with other modalities, such as CITE-seq, was loaded, followed by dimensionality reduction on the entire dataset (23). PCA generated a low-dimensional representation of the data, and uniform manifold approximation and projection (UMAP) was used for visualization. Gene module scores were calculated using the AddModuleScore function to evaluate the relative expression of specific gene sets in each cell, adjusted by subtracting the aggregated expression of a control feature set. In addition, weighted nearest neighbor (WNN) analysis was applied to integrate information from multiple modalities and capture cell-type specific patterns more accurately. The integrated WNN-based representation was visualized using UMAP, and the result was referred to as ‘W UMAP’ to denote the use of the WNN algorithm in the dimensionality reduction process.

### 2.9 Flow cytometry

To separately isolate monocytes, T cells and B cells, frozen PBMCs were thawed and stained with antibodies against surface antigens, after the treatment with FcR blocking reagent (Miltenyi Biotec, Tokyo, Japan). The antibodies used were as follows: CD4-Brilliant Violet 605 (BioLegend), CD20-PE/Cyanine7 (BioLegend), and CD3-Alexa Fluor 488 (BD Biosciences), CD14-Pacific Blue (BD Biosciences), CD19-APC-Cy7 (BD Biosciences). The samples were analyzed on a FACSAria Fusion (BD Biosciences), and monocytes, T cells and B cells were defined as CD3-CD19-CD20-CD14+ cells, CD3+CD4+ cells and CD19+CD20+ cells, respectively.

### 2.10 Quantitative PCR

For the preparation of qPCR analysis, samples were dissolved in ISOGEN II (NIPPON GENE, Tokyo, Japan) kit. The total RNA was reverse transcribed with SuperScript IV VILO Master Mix (Thermo Fisher Scientific, Waltham, MA, USA) according to the manufacturer’s instructions. The qPCR analysis was performed by mixing the TaqMan Fast Advanced Master Mix (Thermo Fisher Scientific, Waltham, MA, USA), TaqMan Gene Expression Assay (Thermo Fisher Scientific, Waltham, MA, USA) and cDNA of each sample. The following TaqMan Gene Expression Assays were used: CX3CR1 (Assay ID: Hs01922583_s1), CCR2 (Assay ID: Hs00704702_s1), CCR5 (Assay ID: Hs99999149_s1), HBA2, HBA1 (Assay ID: Hs00361191_g1), CD180 (Assay ID: Hs00194403_m1), NFKBIA (Assay ID: Hs00355671_g1), DUSP1 (Assay ID: Hs00610256_g1), THBS1 (Assay ID: Hs00962908_m1), TNFAIP3 (Assay ID: Hs00234713_m1), NFIL3 (Assay ID: Hs00705412_s1), CXCL8 (Assay ID: Hs00174103_m1), EGR1 (Assay ID: Hs00152928_m1), SERPINB2 (Assay ID: Hs01010736_m1), and GAPDH (Assay ID: Hs02786624). The results were analyzed with QuantStudio 7 Flex Real-Time PCR System (Applied Biosystems, Waltham, MA, USA). To confirm the significant differences among each sample, Tukey’s multiple comparisons test was applied.

### 2.11 Deconvolution of bulk RNA-seq samples

To determine the proportions of immune cell subsets of bulk RNA-seq data, we employed the CIBERSORTx deconvolution algorithm (https://cibersortx.stanford.edu/) (24). We configured the submitted job type to “Impute Cell Fractions”. The LM22 Signature Matrix file was used as a reference. The relative proportions of immune cells in each dataset were calculated using 1000 permutations, applying B mode batch correction.

## 3 Result

### 3.1 Immune signature changes at NMOSD relapse

To determine whether clinical relapse would affect the gene signature of PBMCs in NMOSD, we compared transcriptome data at several timepoints in a patient in our cohort who underwent relapse during steroid tapering.

**Figure 1:**
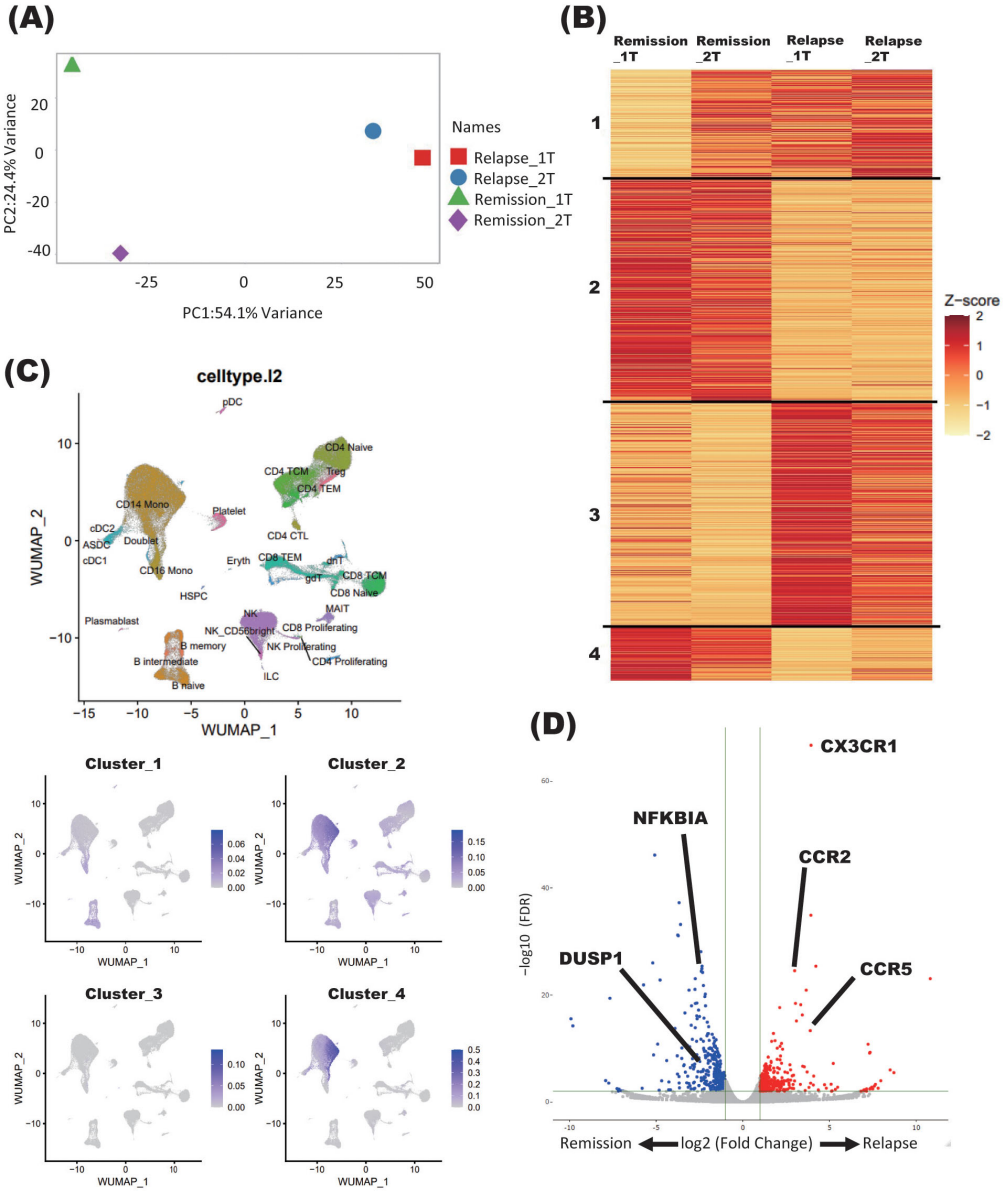
Immune signature changes at the relapse of NMOSD. **(A)** PCA of samples derived from a patient with NMOSD in both remission and relapse phases. Relapse_1T: relapse timepoint; Relapse_2T: after acute-phase treatment; Remission_1T: remission timepoint; Remission_2T: remission timepoint. **(B)** K-means clustering of four samples derived from patients with NMOSD in both remission and relapse phases. Relapse_1T: relapse timepoint; Relapse_2T: after acute-phase treatment; Remission_1T: remission timepoint; Remission_2T: remission timepoint. **(C)** Single-cell annotation of PBMCs, and annotations colored according to cluster expressions determined by K-means analysis of bulk RNA-seq data **(B)**. **(D)** Volcano plot showing DEGs between relapse and remission phases (Relapse v.s Remission).

On the third timepoint of four samples collected, the patient showed relapse and received immunoadsorption therapy. When PCA was performed, the samples obtained at the time of remission and after relapse (in the latter case, both immediately after relapse and after acute-phase treatment) were separated by PC1, indicating 54.1% variance (**Figure 1A**).

Samples obtained during the remission and relapse phases were further analyzed by K-means clustering. Each of the four clusters showed distinct changes in gene signatures before and after relapse. In general, genes comprising clusters 1 and 3 showed enhanced expression at relapse, while those comprising clusters 2 and 4 showed decreased expression (**Figure 1B**).

**Figure 2:**
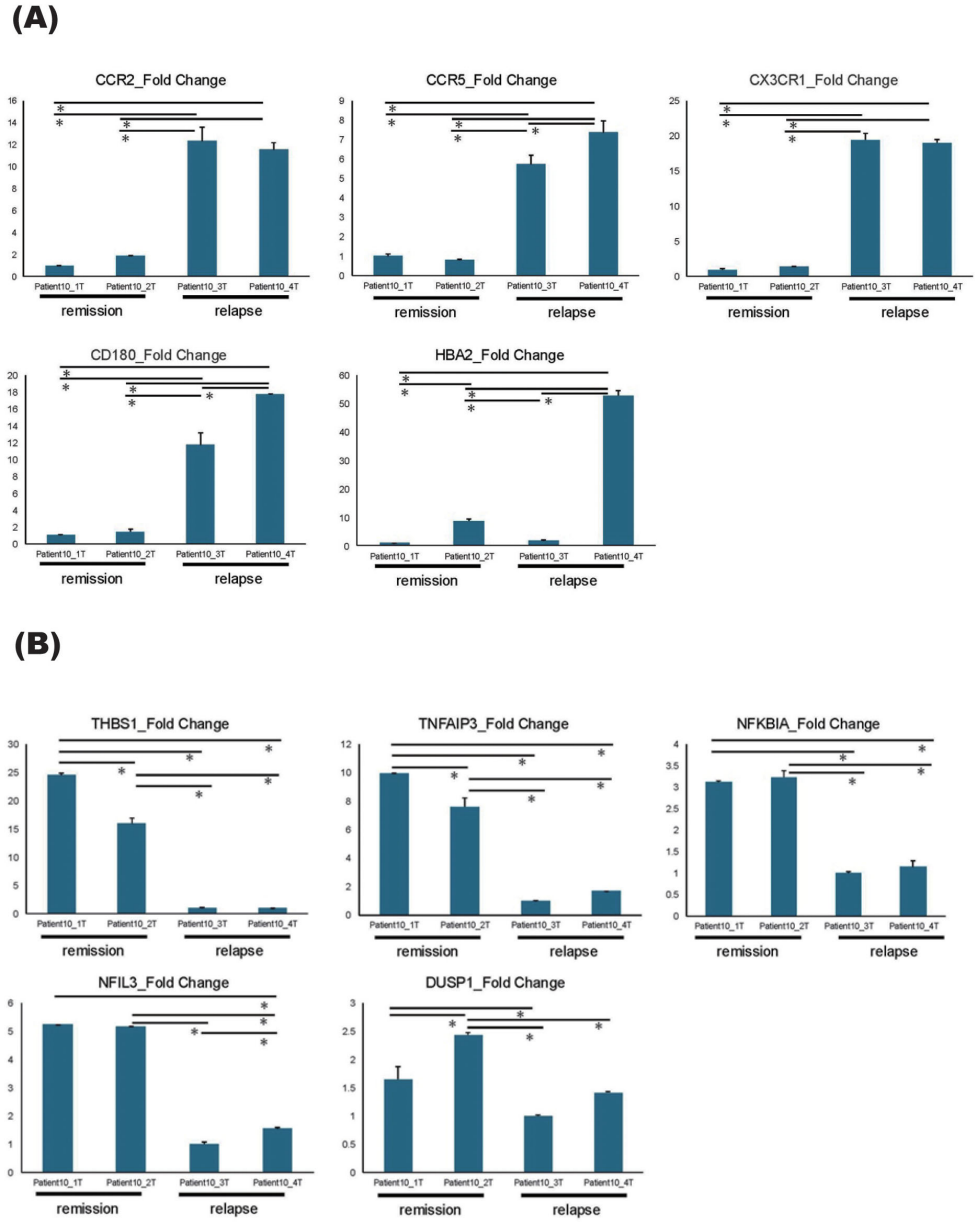
qPCR results of DEGs. **(A, B)** qPCR results of five upregulated genes **(A)** and five downregulated genes **(B)** identified by DEG analysis, comparing the relapse and the remission phases. All samples were collected sequentially over time in the order of their sample numbers. Patient10_1T and Patient10_2T were collected during remission phase, Patient10_3T was collected at relapse timepoint, and Patient10_4T was collected after acute-phase treatment. Error bars represent standard errors (SE). *P < 0.05.

**Figure 3:**
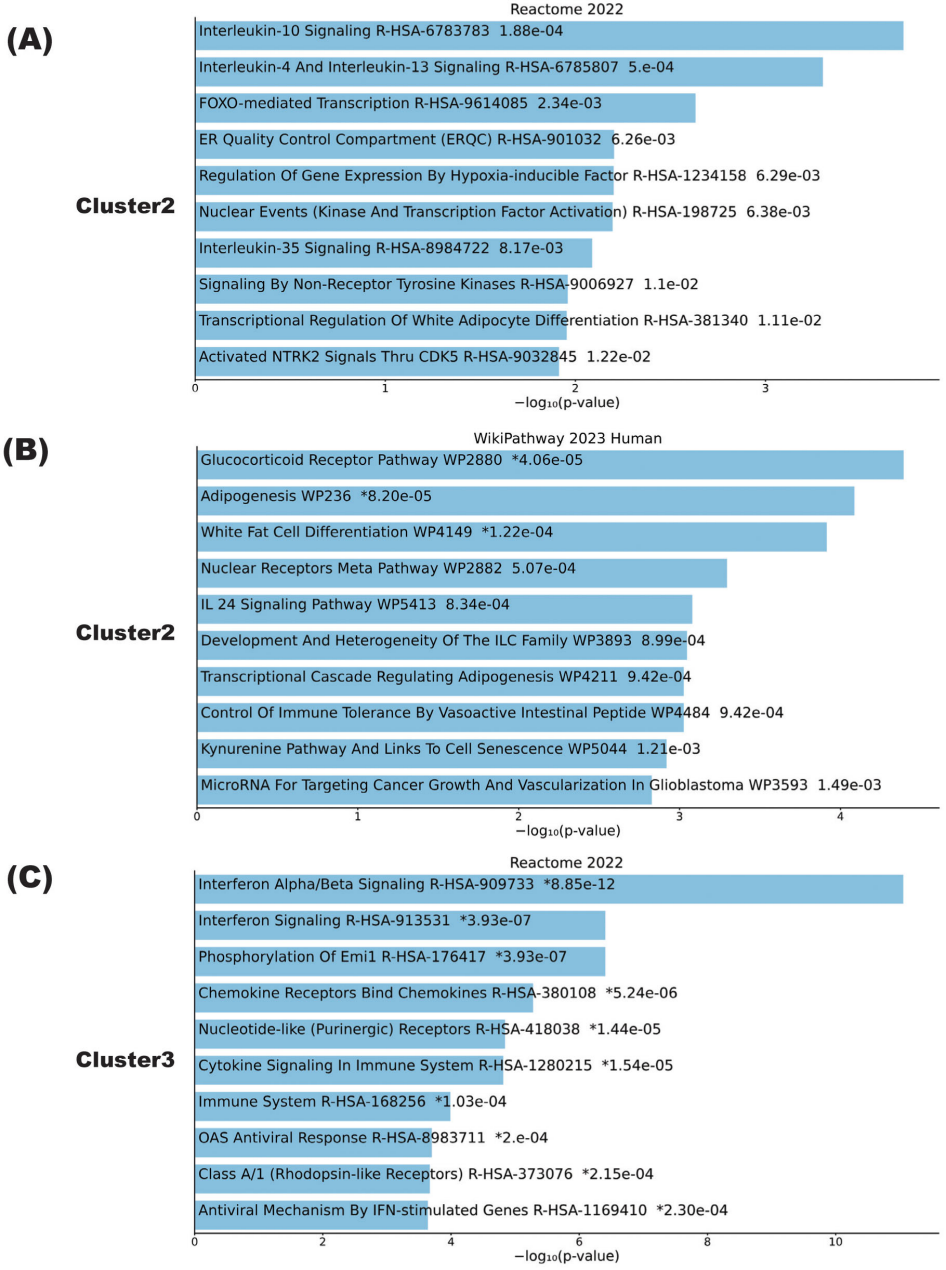
Biological pathways playing central roles in NMOSD relapse. **(A–C)** Pathway analysis results of clusters calculated by K-means in samples from the patient with NMOSD whose data are depicted in **Figure 1**. Cluster pathways shown to be downregulated in the relapse phase according to either the Reactome2022 database **(A)** or the Wikipathway 2023 human database **(B)**. **(C)** Cluster pathways showing enhanced expression in the relapse phase according to the Reactome2022 database. The bar charts show the top 10 most enriched terms, along with their corresponding p-values.

To further elucidate the types of immune cells in which the aforementioned gene signature changes took place, each cluster delineated using the K-means method was applied on single-cell resource of PBMCs (**Figure 1C**). While clusters 1 and 3 did not show specific distributions, both clusters 2 and 4 were predominantly observed in monocyte clusters (**Figure 1C**). In DEG analysis using samples in the relapse and remission phases, immune-related genes such as *CX3CR1* and *CCR2* were upregulated in relapse phases (**Figure 1D**). To validate these results of DEG analysis, qPCR was performed for five upregulated and downregulated genes in the same samples as those used for RNA seq, which were separately aliquoted from the same PBMCs. As a result, we were able to confirm that gene expression patterns were consistent with those observed with DEG analysis (**Figure 2**). It is also important that cluster 2, which was downregulated at relapse, was characterized by both interleukin 10 (IL-10) signaling and glucocorticoid receptor pathways (**Figure 3**). Moreover, cluster 3, which was characterized by enhanced gene expression at relapse, showed an association with the interferon pathway (**Figure 3**). Similar results were observed for cluster 4, which was also downregulated at relapse (data not shown).

### 3.2 Transcriptome signatures of PBMCs in patients with steroid tapering

**Figure 4:**
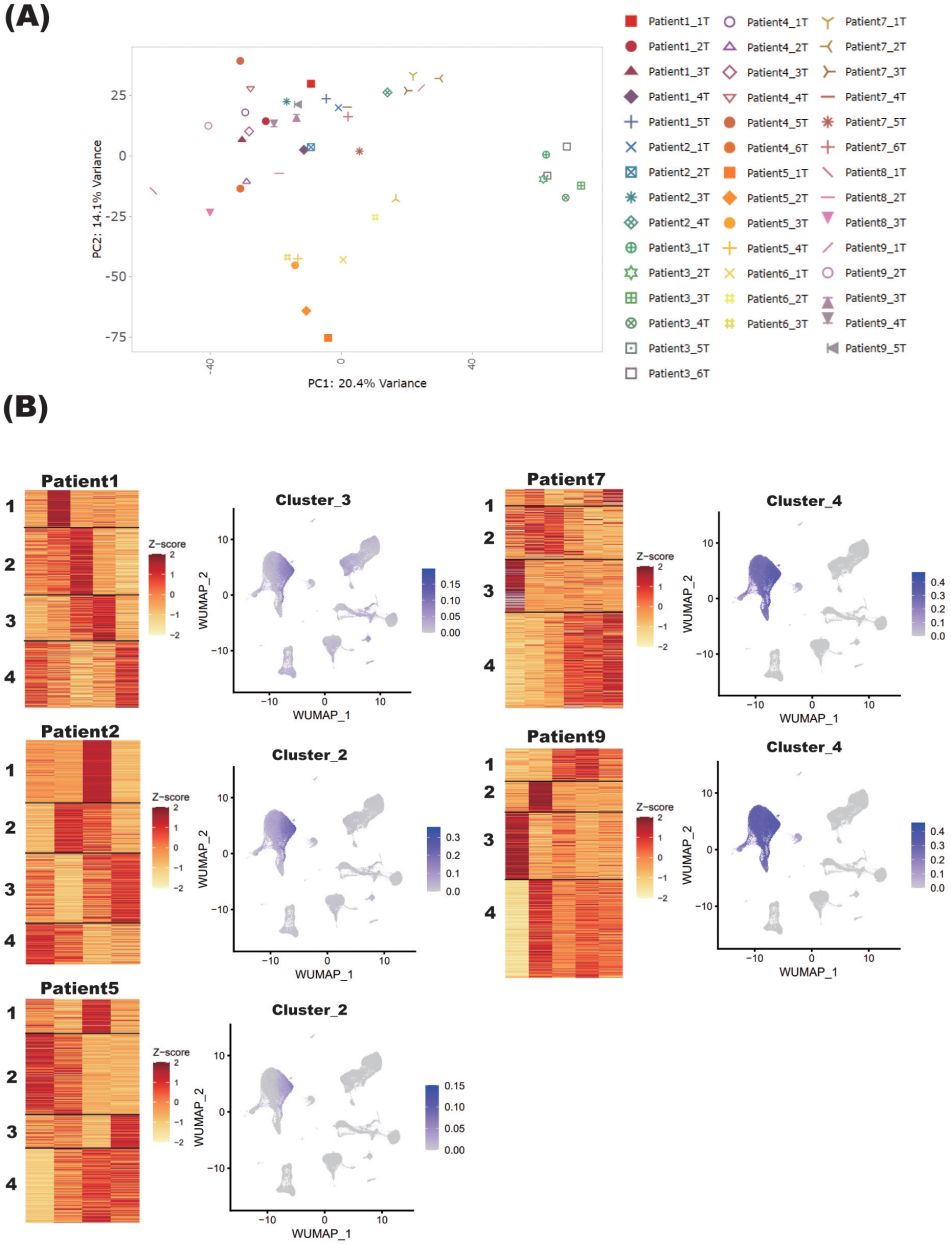
Blood transcriptome signature of patients with NMOSD under steroid tapering. **(A)** PCA of blood samples from nine patients with NMOSD. Sample names refer to the annotated patient number and the chronological timepoints at which the samples were collected, the latter described by “T” in numerical order. **(B)** For Patients 1, 2, and 5 (Group 1), and Patients 7 and 9 (Group 2), heatmaps under steroid tapering are shown, along with single-cell graphs of PBMCs colored according to the expression of genes in each cluster. For each patient, steroid dose decreases from left to right on the heatmaps.

To further comprehend the sample variance depending on the status of steroid tapering, all the samples collected from 9 individual patients at different steroid dosage were analyzed for PCA analysis. The PC1 and PC2 variances were 20.4% and 14.1%, respectively, and the samples tended to be grouped according to patient identity rather than steroid dosage (**Figure 4A**).

To determine how gene signatures were related to steroid dosage, transcriptome data of samples collected from each patient were separately analyzed by the K-means method. Although the cohort as a whole did not exhibit a consistent pattern, three clustering types could be defined on the basis of the gene clustering patterns in monocytes. Specifically, in Patients 1, 2, and 5 (Group 1), the gene sets reduced by steroid tapering were highly expressed in monocytes (**Figure 4B**). By contrast, in Patients 7 and 9 (Group 2), those upregulated by steroid tapering were also highly expressed in monocytes (**Figure 4B**). Other clusters were randomly distributed across non-specific cell populations (**Supplementary Figure S1**). Of note, genes with upregulated expression in Group 2 were associated with inflammatory pathways, especially interferon signaling, and those with downregulated expression were enriched in anti-inflammatory and glucocorticoid receptor pathways, as represented by IL-10 signaling (**Supplementary Figures S2**, **S3**). These varied observations among patients were not unexpected given their differing immunological statuses. The important thing is that patients who showed similar downregulation of genes associated with anti-inflammatory and glucocorticoid receptor pathways might have similar transcriptomic changes in the relapse phase, and represent the preconditioned status of NMOSD that is at the edge of reactivation while steroid is being tapered.

**Figure 5:**
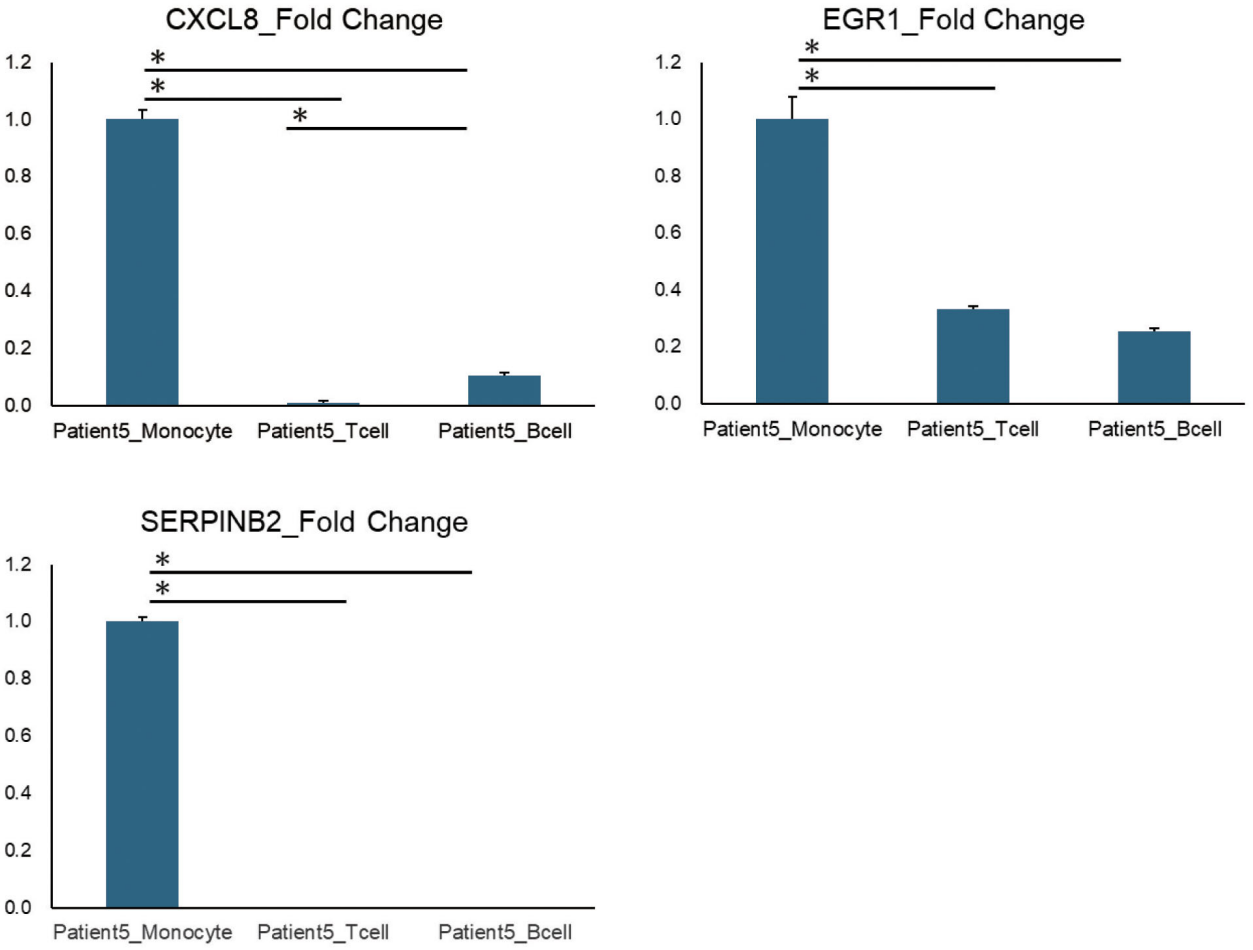
qPCR results of representative genes identified by K-means method. The qPCR results of representative genes (CXCL8, EGR1, and SERPINB2) listed in Cluster 2 of Patient 5, identified using K-means method. Patient5_Monocytes, T cells, and B cells refer to three types of immune cells (monocytes, T cells, and B cells) that were isolated from the PBMCs of Patient 5 using flow cytometry. Error bars represent standard errors (SE). *P < 0.05.

To validate whether the genes in the clusters obtained by the K-means method were actually expressed predominantly in a specific cell subtype, we have separately isolated CD14+ monocytes, CD4+ CD3+ T cells and CD20+ CD19+ B cells by flow cytometry and performed qPCR analysis to compare the gene expression of the interest. Although due to the limited amounts of residual samples which were aliquoted from the same PBMCs as used for RNA-seq analysis we were not able to perform the validations for the whole results, we were able to confirm that genes included in the cluster 2 of Patient 5 which were assigned to monocytes were significantly highly expressed in monocytes by qPCR analysis (**Figure 5**).

Moreover, by applying deconvolution techniques of CIBERSORT we confirmed specific cell subtypes which were identified by gene set scoring in single-cell RNA-seq dataset. The deconvolution results of cluster 4 of patient 9, identified by K-means method, were shown to be as follows: the percentages of monocyte, macrophage, and dendritic cell populations were more predominantly expanded in the sample such as Patient9_5T, where steroid dosage was most reduced (**Supplementary Figure S4**).

In addition, as control cohort, we utilized bulk RNA-seq data of PBMCs derived from three patients positive for anti-acetylcholine receptor antibody. K-means clustering and gene set scoring in single-cell RNA-seq dataset were performed following the same processes as those for NMOSD samples (**Supplementary Figure S5**). In control samples, the genes in each cluster were predominantly distributed either to B cells or to NK cells, which pattern was remarkably distinct from those of NMOSD samples, where monocyte distribution has been their characteristic results.

### 3.3 Chronological landscape of relapse-related clusters in patients with steroid tapering

Since a relapsed patient exhibited distinct gene clusters showing either upregulated or downregulated gene expression (**Figure 1B**), we sought to clarify the chronological landscape of each cluster in patients undergoing steroid tapering. Certain trend was observed with cluster 2, which showed downregulation at relapse, in patient 1, 2, 5 and 6 (**Supplementary Figure S6**). By contrast, in Patients 5 and 6, cluster 3 exhibited enhanced expression at relapse, and this expression was positively correlated with steroid tapering (**Supplementary Figure S7**). These observations suggest that gene signatures observed during flares of disease activity might also be present in the PBMCs of patients undergoing steroid tapering.

### 3.4 Steroid-related WGCNA modules in NMOSD

**Figure 6:**
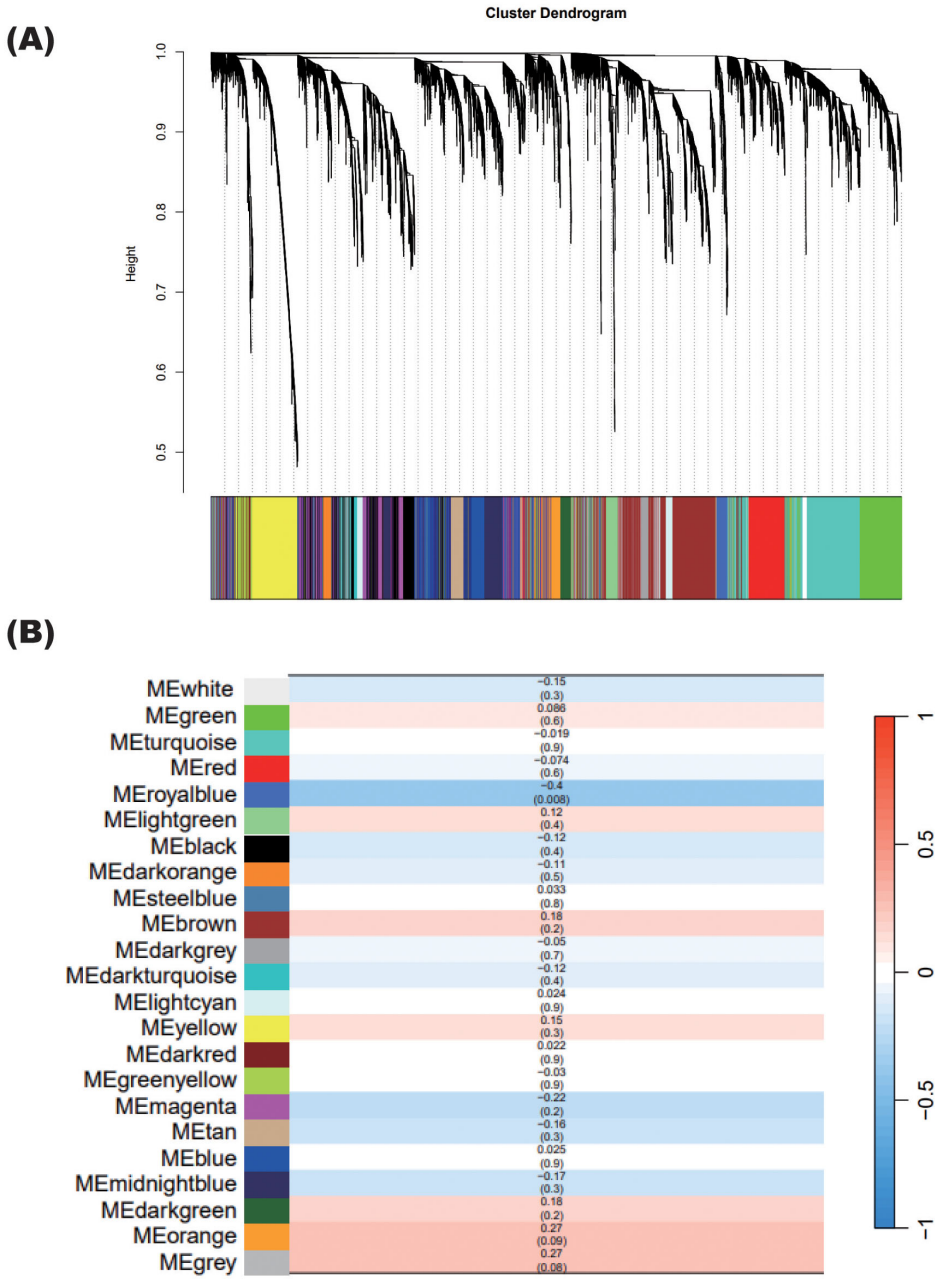
WGCNA showing specific modules correlated with steroid tapering. **(A)** Gene cluster dendrogram. Each branch in the figure represents one gene, and each color represents the corresponding co-expression module. **(B)** Correlations between each module calculated in **(A)** and the steroid dosages of patients with NMOSD, excluding the relapsed patient. The numbers in each cell show the correlation (upper) and the p-value (lower).

As gene expression was not significantly consistent between patients undergoing steroid tapering, as mentioned above, we used WGCNA to identify unbiased gene modules related to steroid dosing. WGCNA revealed 23 modules among the nine patients other than the one who experienced relapse (Patient 10) (**Figure 6A**).

Only the royalblue module was significantly correlated with steroid dosage, with a correlation coefficient of 0.4 (**Figure 6B**). The correlation coefficient value was negative, indicating that the expression levels of genes in the royalblue module increase when the steroid dosage decreases.

**Figure 7:**
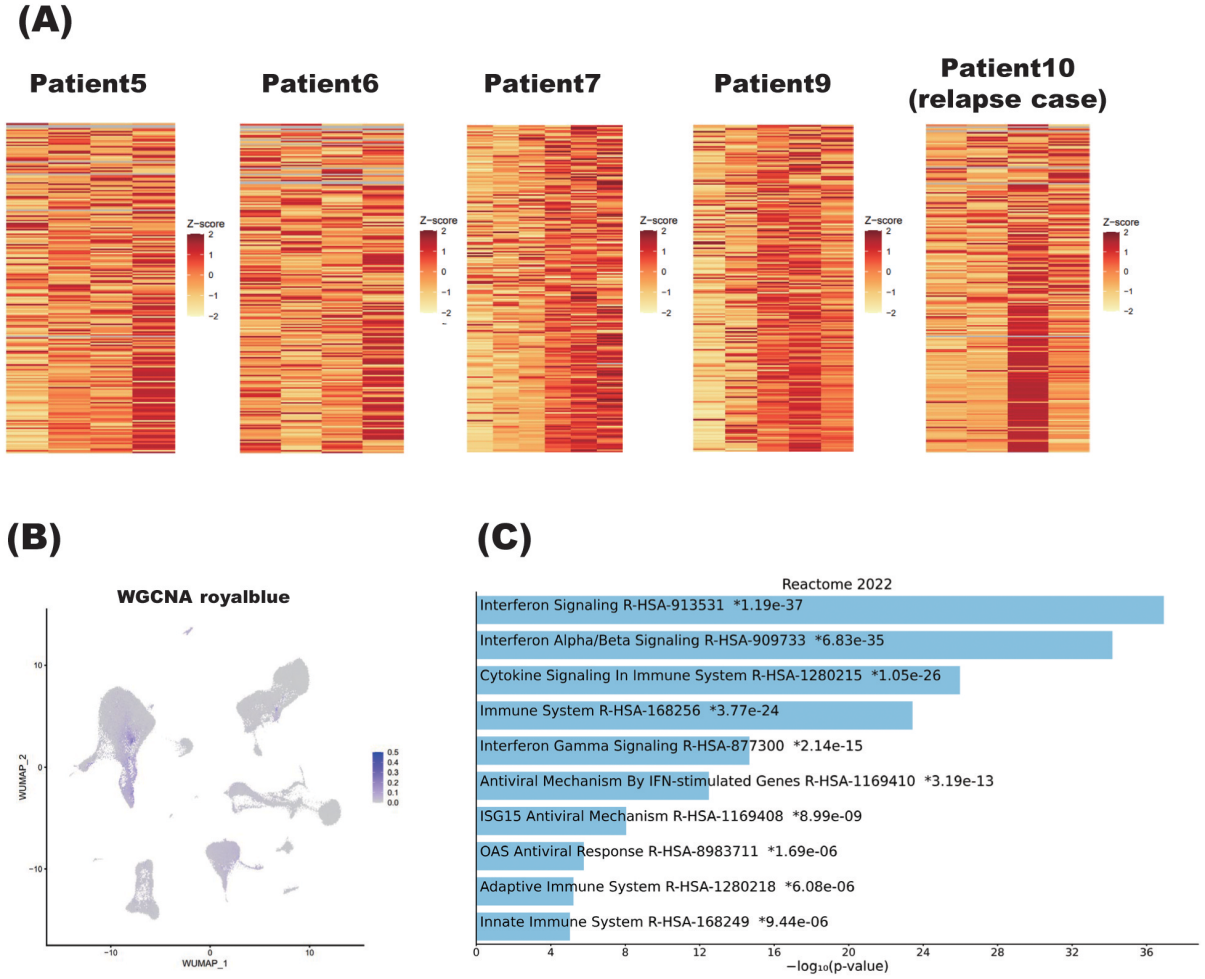
Characteristics of steroid dosage correlated with weighted gene co-expression network analysis (WGCNA) module. **(A)** Heatmaps showing the correlation between the royalblue module, identified by WGCNA, and steroid tapering in patients with NMOSD. For Patients 5, 6, 7, and 9, the steroid dose decreases from left to right on the heatmaps. Patient 10 is the individual who relapsed, and the two left columns and two right columns represent samples obtained during the remission and relapse phases, respectively. More specifically, within the two right columns, the left column represents the sample obtained immediately after relapse, while the right column represents the sample immediately after acute-phase treatment. **(B)** Single-cell graphs of PBMCs colored according to the expression of the royalblue module identified by WGCNA as correlating with steroid tapering. **(C)** Pathways of modules identified by WGCNA as correlating with steroid tapering according to the Reactome2022 database. The bar chart shows the top 10 most enriched terms along with their corresponding p-values. .

After identifying this steroid-correlated module, we examined how its associated genes changed in each patient as the steroid dosage was reduced (**Figure 7A**). Importantly, genes in the royalblue module showed remarkable enhancement at relapse in Patient 10. Given that this module was identified in the group of patients excluding Patient 10, it is interesting that the gene signature associated with steroid tapering was apparent in this case of relapse.

As for the other nine patients undergoing steroid tapering, Patients 5, 6, 7, and 9 showed enhanced expression of royalblue module genes. Royalblue module genes were expressed predominantly in the monocyte subset, although this preferential expression was rather subtle (**Figure 7B**).

To confirm specific cell subtypes which were identified by gene set scoring in single-cell RNA-seq dataset, we have validated the results by applying deconvolution techniques of CIBERSORT. The results of the deconvolution for WGCNA royalblue module were assigned to monocyte, macrophage, and dendritic cell populations. For example, in patient 5, the percentage of monocytes, macrophages and dendritic cells was shown to be more abundant in Patient5_4T sample, where steroid dosage was most reduced, compared to samples collected at other timepoints (**Supplementary Figure S8**).

Importantly, pathway analysis showed that royalblue module genes were associated with the interferon pathway (**Figure 7C**). In the royalblue module, we identified genes important for the inflammatory response, including interferon-related genes and those involved in T cell and B cell differentiation (**Table 3**).

**Table 3:** Gene list in royalblue module.

IFITM3	ISG15	PSME2	IFITM1	IFI6	IFITM2
IFI27	LY6E	PSME1	PSMB9	UBE2L6	BST2
OAS1	STAT1	EIF5A	MX1	FOLR3	TAP1
LGALS3BP	ISG20	IFIT3	TRIM22	IRF7	LAP3
XAF1	SHISA5	IRF9	IFI35	PARP9	SP100
IFI44L	OAS2	PLSCR1	PLAC8	IFI44	IFI16
GBP1	IFIT2	SERPING1	EPSTI1	USP18	IDH2
CMPK2	OASL	RABGAP1L	TRIM5	TRANK1	WDR86-AS1
NMI	MX2	PLD4	SPATS2L	ST3GAL5	FAM13A
OAS3	ADAR	RSAD2	PPM1K	SETX	PI4K2B
GBP5	PARP10	SP110	LILRA4	DDX11	CYB561
EAF2	CCDC167	STAT2	TRIM25	RMI2	SMG7
PHF11	HSH2D	PARP12	MT1L	IGFBP4	GCNT1
SIGLEC1	SAMD9L	IFIT1	LINC00467	CD70	CD2AP
JUP	HERC5	TIMM10	NFE2L3	P2RY6	CCDC50
FBXO6	ARF6	LCP2	CDKN2A	N4BP1	RUFY4
PARP14	ZBP1	HERC6	IL12RB2	SOS1	P2RY11
CHMP5	CXCL10	DDX60	GBP1P1	IFNG-AS1	RTN4IP1
CUL1	RNASEH2B	DDX60L	UHRF1	WASH5P	EDARADD
DDX58	HLA-DQB2	ACAD9	MSL3P1	SCD	SNX7
LAG3	EIF2AK2	TIGIT	KMO	HELZ2	SAMD4A
GBP4	SP140	MOV10	FOXP3	TRIP6	NDUFC2-KCTD14
RNF213	OTOF	HYPK	KCNMA1	CLEC4C	USP12-AS1
CMC2	IFIH1	PML	TARM1	ACY3	AXL
DHX58	MT1E	DTX3L	MACROD2	LHB	CDCA3
DUT	SAMD9	XAB2	CHAD	LINC01013	SPSB1
UBE2L3	CMTR1	KHDRBS1	DEFB1	HESX1	CDT1
TFDP1	ANGPTL6	NUB1	IGFL2	CCL23	FAM111B
BISPR	DUSP5	DCAF11	PACSIN1	GNG3	APOBEC3B-AS1
MTHFD1	GTSF1	THRAP3	S1PR2	SCT	OR56B1
ETV7	IFIT5	ZNFX1	ACE	PIP5K1B	SPACA3
DDB2	REC8	CPT1B	CXCL11	QSER1	L1TD1
POP4	RNF31	UBE2Z	HRK	CATSPERG	MAGOH2P
SLC38A5	USP30-AS1	UPK3A	PLA2G2D	SOGA3	OR2L2
IL4I1	APOL6	APOBEC3H	KIF20A	SYT15	PTPN13
BAZ1A	TDRD7	LGMN	S100A3	S100A1	BRCA2
KLHDC7B	ANKFY1	UTRN			

All of these observations suggest the presence of a steroid-related gene module in PBMCs, and indicate that the innate pathway, as represented by the interferon signature, might represent fluctuating NMOSD activity during steroid tapering.

## 4 Discussion

The complete prevention of relapses and appropriate therapeutic strategy of NMOSD has become nearly feasible with the advent of various monoclonal antibody (25), such as eculizumab, satralizumab, inebilizumab, and rituximab. However, attaining the attractive treatment goal can only be met with monitoring the disease activity of NMOSD (26–28); in this regard, several issues must still be addressed. One of these is the development of steroid tapering regimens that prevent steroid-related side effects (29, 30) and clinical relapses. Our study therefore analyzed the blood of NMOSD patients with the goal of identifying an unbiased transcriptome signature that can serve as a novel disease marker.

Monocytes have been shown to play a pivotal role in the pathogenesis of various autoimmune diseases (31, 32). Monocytes are immune cells that originate from the bone marrow, and are released into the peripheral blood (33). Five to ten percent of peripheral blood leukocytes are monocytes; they belong to the mononuclear–phagocyte system, which includes macrophages, dendritic cells, and monocyte precursors (33). Monocyte activation is associated with the progression of autoimmune diseases such as systemic lupus erythematosus (SLE) (31, 34) and rheumatoid arthritis (RA) (32, 35). In SLE, the non-classical monocyte subset is associated with autoantibody production and antigen-presenting capability, while in RA, the non-classical subset has been shown to differentiate into inflammatory monocytes, and this process is associated with osteoclasts (36).

Notably, in the one patient in this study who experienced clinical relapse, the transcriptomic change in PBMCs represented a dynamic change in the immune signature. The relapse-associated genes were related to the interferon, virus-related, and chemokine receptor pathways. These observations are consistent with previous studies showing the pivotal role of the interferon pathway in the NMOSD immune signature (37, 38). PBMCs isolated from patients with NMOSD show a high expression of type 1 interferon, which is thought to be potentiated by the stimulation of plasmacytoid dendritic cells exposed to cell-free DNA released by NETosis (39). Clinically, it is well recognized that NMOSD show concomitant presence of type 1 interferon-related diseases, such as SLE or Sjogren syndrome at high frequencies (40). Moreover, administration of recombinant interferon is reported to have induced the clinical attack of NMOSD (41), and steroid is shown to inhibit the augmentation of interferon pathway (42). The above-mentioned critical role of interferon pathway in the pathogenesis of NMOSD is further elucidated by utilizing the mice deficient for interferon receptor (43).

It is interesting to note that the relapsed patient in this study showed downregulation of the glucocorticoid receptor pathway. This observation further supports the notion that this pathway plays major roles in inhibiting NMOSD disease activity. Our study also clarified the central role of IL-10. It is known that stimulation with CD40L results in decreased IL-10 production in classical monocytes positive for the surface markers CD14 and CD16 (i.e., CD14^++^CD16^+^) compared with healthy controls, suggesting the involvement of these monocytes in the decreased serum IL-10 levels in NMOSD (44). Therefore, decreased numbers of anti-inflammatory monocytes might represent augmented NMOSD disease activity.

In this study, DEG analyses performed both at relapse and remission detected monocyte-related genes such as *CX3CR1* and *CCR2*. CCR2 is a receptor for CCL2 (45), which is a CC chemokine ligand, and is known to be involved in inflammation and demyelination in the central nervous system (46, 47). CX3CR1 is a receptor for CX3CL1, also called fractalkine, and is involved in the interaction between neurons and microglia (48). It has previously been shown that CX3CR1 expression is upregulated in monocytes in NMOSD, which is consistent with our observations (49). With regard to other genes, it is interesting that *DUSP1* and *NFKBIA* were downregulated at the time of relapse. DUSP1 inhibits the MAPK signaling pathway, particularly through pathways involving JNK (c-Jun N-terminal kinase) and p38 (50). NFKBIA, on the other hand, inhibits the NF-κB pathway, and is a member of the inhibitor of NF-κB (IκB) family (51). MAPK signaling pathway plays major role in the production of IL-β, and NF-κB pathway in IL-β and IL-6, respectively (52, 53). IL-β and IL-6 is recognized as key cytokines in the pathogenesis of NMOSD, such as differentiation of proinflammatory Th17 cells and survival of B cell population. Therefore, the fact that these two genes were downregulated at the time of relapse might reflect the underlying inflammatory pathway in NMOSD during a disease flare.

The effects of steroids, especially glucocorticoids, on monocytes include suppression of the release of pro-inflammatory mediators such as IL-1β and IL-6, and promotion of the release of anti-inflammatory mediators such as IL-10 and TGF-β (54). In addition, it has been reported that monocytes treated with glucocorticoids exhibit improved phagocytosis, anti-apoptotic effects, and enhanced migration (55). Consequently, glucocorticoids induce differentiation of monocytes into a subtype that may have anti-inflammatory effects. In this study, it was particularly intriguing to observe the decrease in anti-inflammatory monocytes following steroid tapering in several patients, and similar immune dynamics in monocytes at the relapse of NMOSD. This suggests that increased NMOSD disease activity may be attributed to the onset of monocyte signature changes.

Using WGCNA, we found that in several patients, steroid tapering was associated with the upregulated expression of genes in the royalblue module, and a similar pattern was observed in the relapsed patient. Several genes included were those related to interferon, such as *IFITM1*, *IFITM3*, *ISG15*, *STAT1*, *MX1*, and *OAS1* (56–60), as well as those related to T cell and B cell differentiation, such as *LY6E* and *BST2* (61, 62). These genes are believed to be upregulated in association with inflammation. Interestingly, a previous study demonstrated a relationship between monocytes and interferon; specifically, the recruitment of monocytes to inflammatory sites was facilitated by MCP-1 (monocyte chemoattractant protein-1), which is induced by interferon-α (63).

This study has three main limitations. First, the Seurat package, which we used to identify cell populations, utilizes single-cell reference data. Therefore, the cell populations obtained from bulk RNA-seq data using Seurat may exhibit slight discrepancies. Second, in cluster-based temporal analysis, the potential impact of external factors was not completely excluded, thus leaving the possibility that gene expression patterns might have partially been affected. Third, due to the considerably rare prevalence and incidence of the disease, the cohort of this study was limited to small number of patients, and there were variations in the types and dosages of immunosuppressive drugs co-administered to different patients and heterogeneity of clinical background, which may have influenced the results. Based upon the same reason, clinical variability such as disease duration, prior treatment history and other comorbidities might have affected in ensuring comparability among patients. In the future, we would like to accumulate more cases and conduct analyses in which there is uniformity of concomitantly administered immunosuppressive drugs and homogenous clinical background. Additionally, in this study only Japanese patients were included, thus requiring populations of various racial background to lead more generalized conclusion of our findings.

This study is the first to analyze the transcriptome signature of PBMCs obtained from the blood of NMOSD patients, with the aim of identifying novel disease markers in this condition. Some of the gene sets that exhibited differences during relapse and steroid tapering were monocyte related, with functionality related to increased inflammatory activity and decreased anti-inflammatory activity. Furthermore, WGCNA identified a module whose component genes showed expression levels that were inversely correlated with steroid reduction, and the entire module was upregulated at the time of relapse. These findings, together with the results of larger cohort analyses in the future, will facilitate the development of novel strategies to achieve more personalized regimens for the treatment of NMOSD. In the future, by monitoring chronological change in the expression of the gene sets that were shown to correlate with immune flare or steroid reduction in our study, the decision making of the speed of steroid reduction and the amount of the steroid required for the maintenance would become feasible in patients with NMOSD who are either on concomitant biologics or on steroid-centered treatment.

## Data Availability

The datasets presented in this study can be found in online repositories. The names of the repository/repositories and accession number(s) can be found in the article/**Supplementary Material**.

## Supplementary material

Supplementary Figure 1 Single-cell graphs representing each cluster in Patients 1, 2, 5, 7, and 9.

Supplementary Figure 2 Pathway analysis of each cluster in Patients 1 and 2. For Patients 1 and 2, pathway analysis was performed according to either the Reactome2022 database (top) or the Wikipathway 2023 human database (bottom). The bar charts show the top 10 most enriched terms along with their corresponding p-values.

Supplementary Figure 3 Pathway analysis of each cluster in Patients 5, 7, and 9. For Patient 5, pathway analysis was performed according to either the Reactome2022 database (top) or the Wikipathway 2023 human database (bottom). For Patients 7 and 9, pathway analysis was performed according to the Wikipathway 2023 human database. The bar charts show the top 10 most enriched terms along with their corresponding p-values.

Supplementary Figure 4 CIBERSORT analysis of the cluster identified by K-means method. The deconvolution results for cluster 4 of patient 9 identified by K-means method. Patient9_1T to Patient9_5T samples were chronologically collected according to steroid dosage.

Supplementary Figure 5 Control sample analysis. **(A, B)** Heatmap **(A)** and single-cell graphs of PBMCs colored according to the expression of genes **(B)** in each cluster identified using K-means method in three control samples. Control_1, Control_2 and Control_3 represent patients positive for anti-acetylcholine receptor antibody.

Supplementary Figure 6 Chronological changes in the cluster downregulated at NMOSD relapse in patients under steroid tapering. The relative expression of cluster 2, which was downregulated at NMOSD relapse, is depicted chronologically in patients under steroid tapering. The steroid dose decreases from left to right in the graphs. The line in each graph indicates the mean value, with the range from −2SD to +2SD highlighted in gray.

Supplementary Figure 7 Chronological changes in the cluster upregulated at the NMOSD relapse in patients under steroid tapering. The relative expression of cluster 3, which was upregulated at NMOSD relapse, is depicted chronologically in patients under steroid tapering. The steroid dose decreases from left to right in the graphs. The line in each graph indicates the mean value, with the range from −2SD to +2SD highlighted in gray.

Supplementary Figure 8 CIBERSORT analysis of WGCNA royalblue module. The deconvolution results for WGCNA royalblue module in patient 5. Patient5_1T to Patient5_4T samples were chronologically collected according to steroid dosage.
