# Supplementary material for: Transcriptome signature in the blood of neuromyelitis optica spectrum disorder under steroid tapering
Source: Front Immunol. 2025 Feb 3;16:1508977. doi: 10.3389/fimmu.2025.1508977 (PMC11830620; doi:10.3389/fimmu.2025.1508977)
Supplement: Supplementary Figure 1 — Single-cell graphs representing each cluster in Patients 1, 2, 5, 7, and 9. [file Presentation1.pptx]

## Slide 1
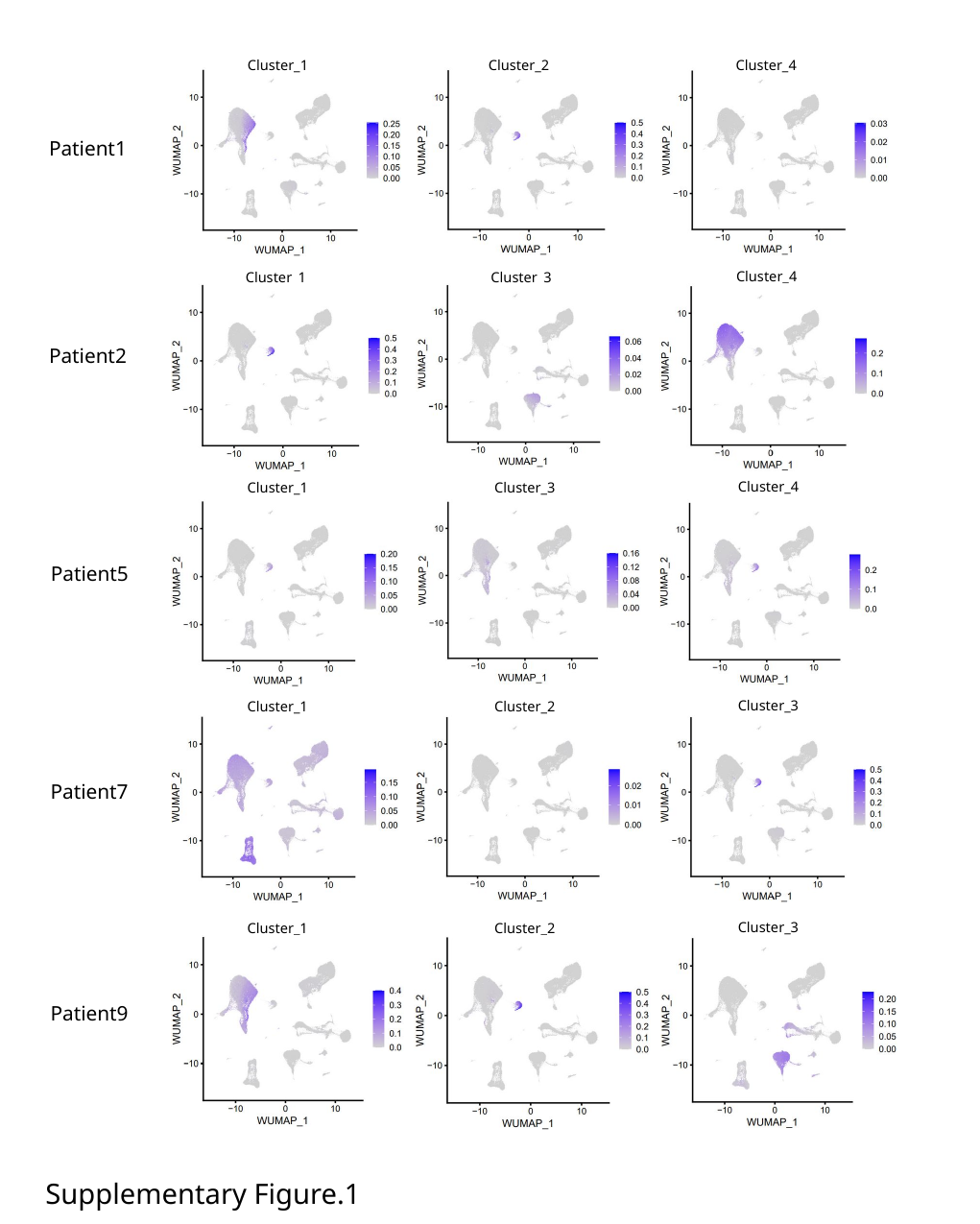

Cluster_2
Cluster_4
Cluster_1
Patient1
Cluster_4
Cluster_1
Cluster_3
Patient2
Cluster_4
Cluster_3
Cluster_1
Patient5
Cluster_3
Cluster_2
Cluster_1
Patient7
Cluster_3
Cluster_2
Cluster_1
Patient9
Supplementary Figure.1

## Slide 2
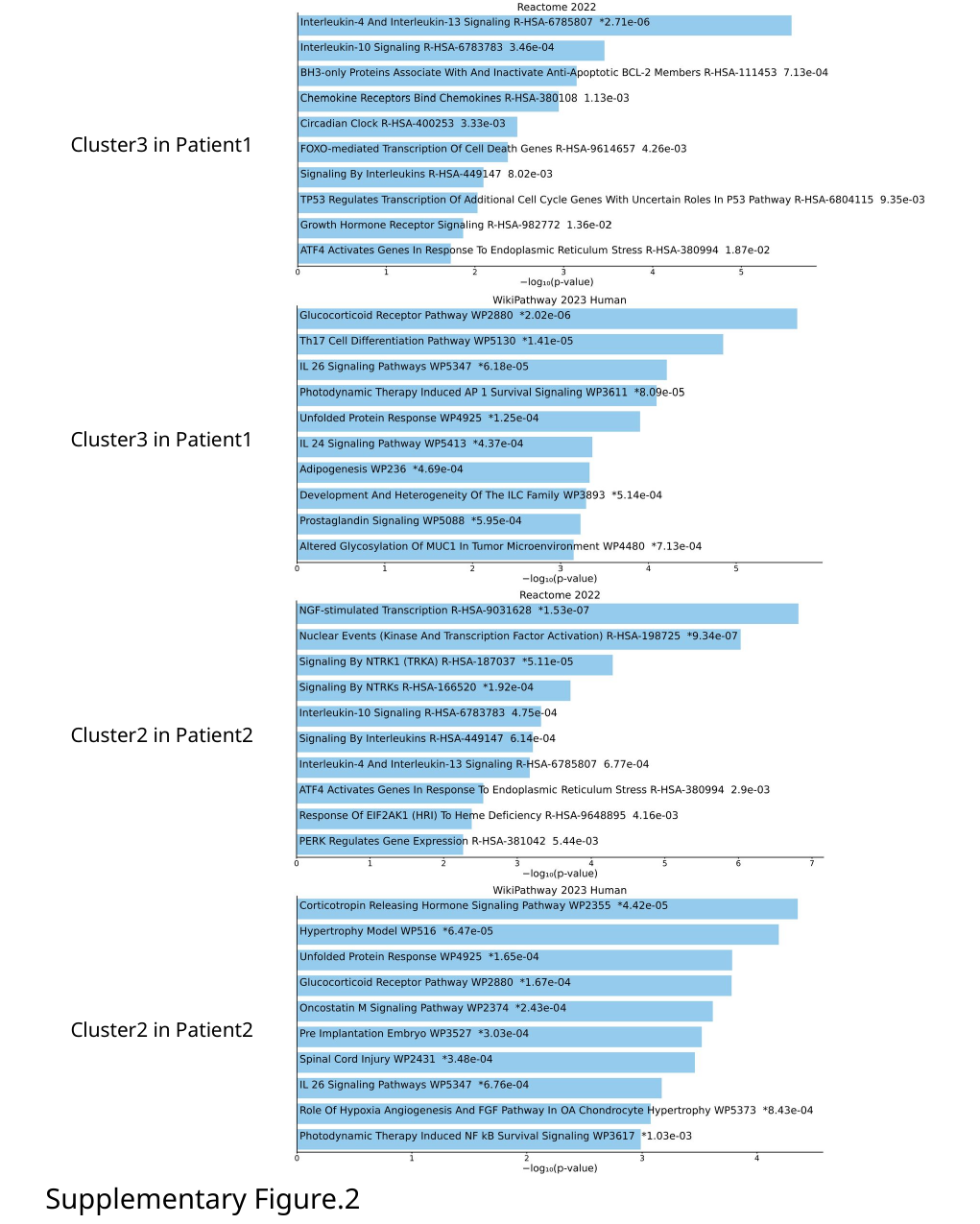

Cluster3 in Patient1
Cluster3 in Patient1
Cluster2 in Patient2
Cluster2 in Patient2
Supplementary Figure.2

## Slide 3
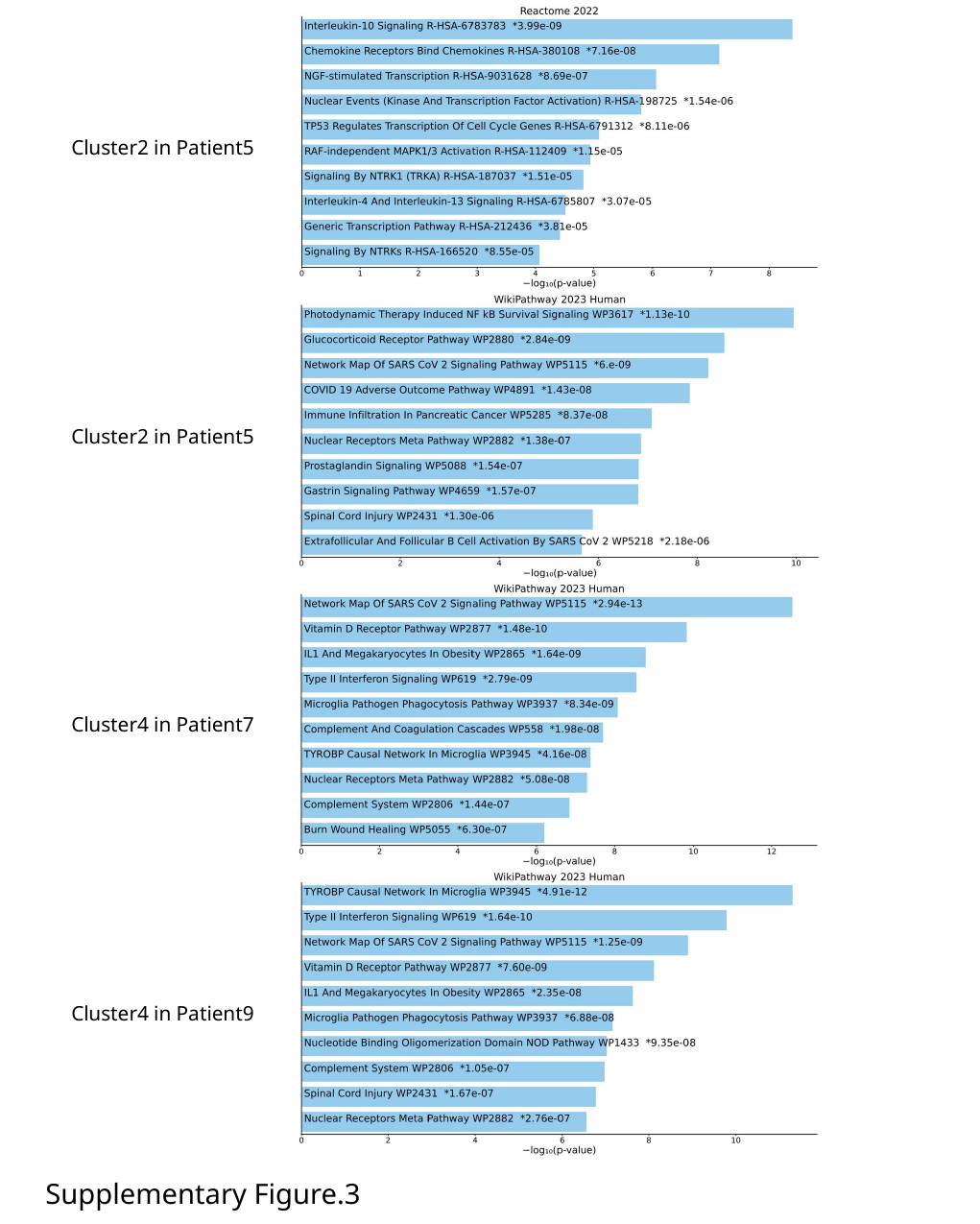

Cluster2 in Patient5
Cluster2 in Patient5
Cluster4 in Patient7
Cluster4 in Patient9
Supplementary Figure.3

## Slide 4
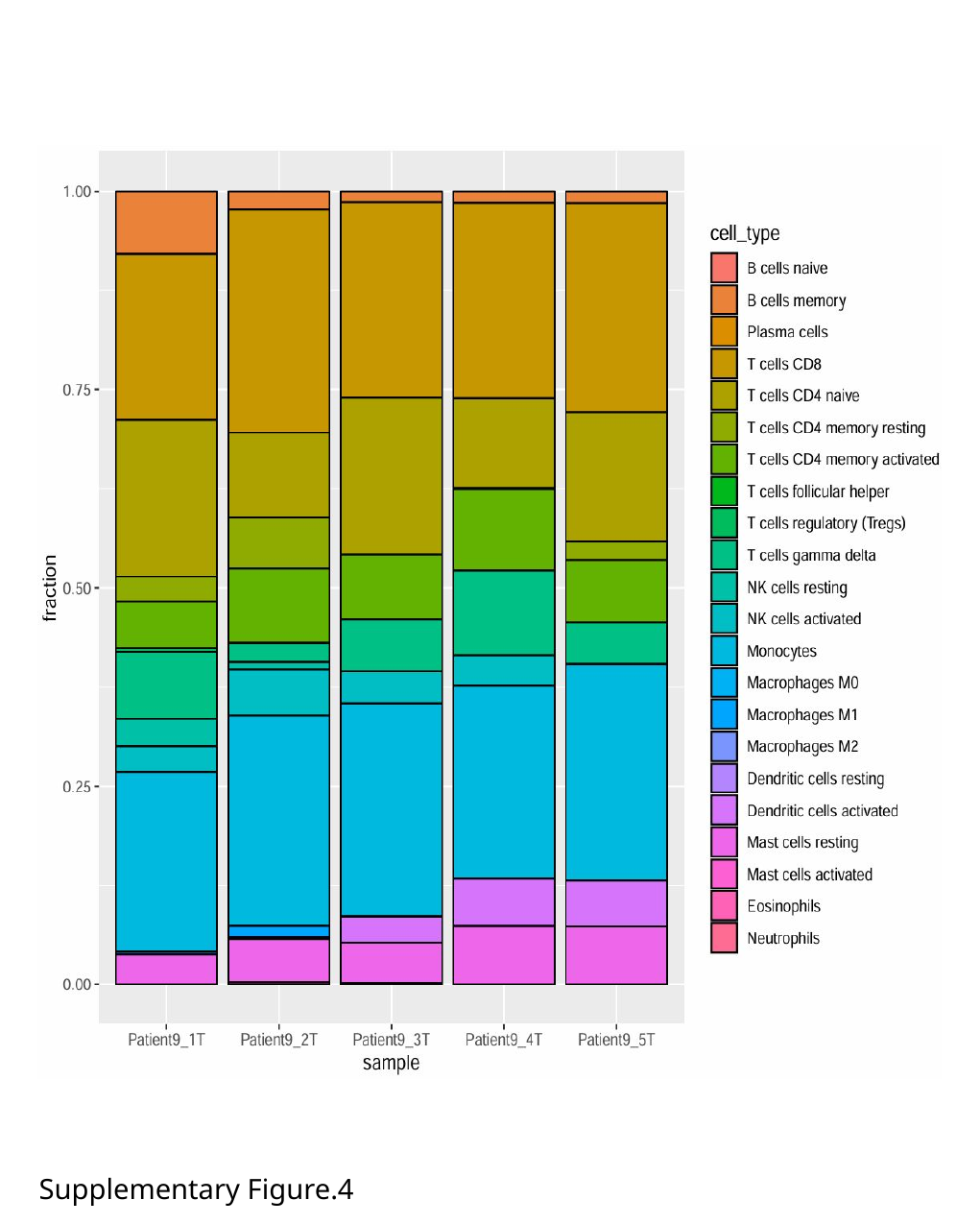

Supplementary Figure.4

## Slide 5
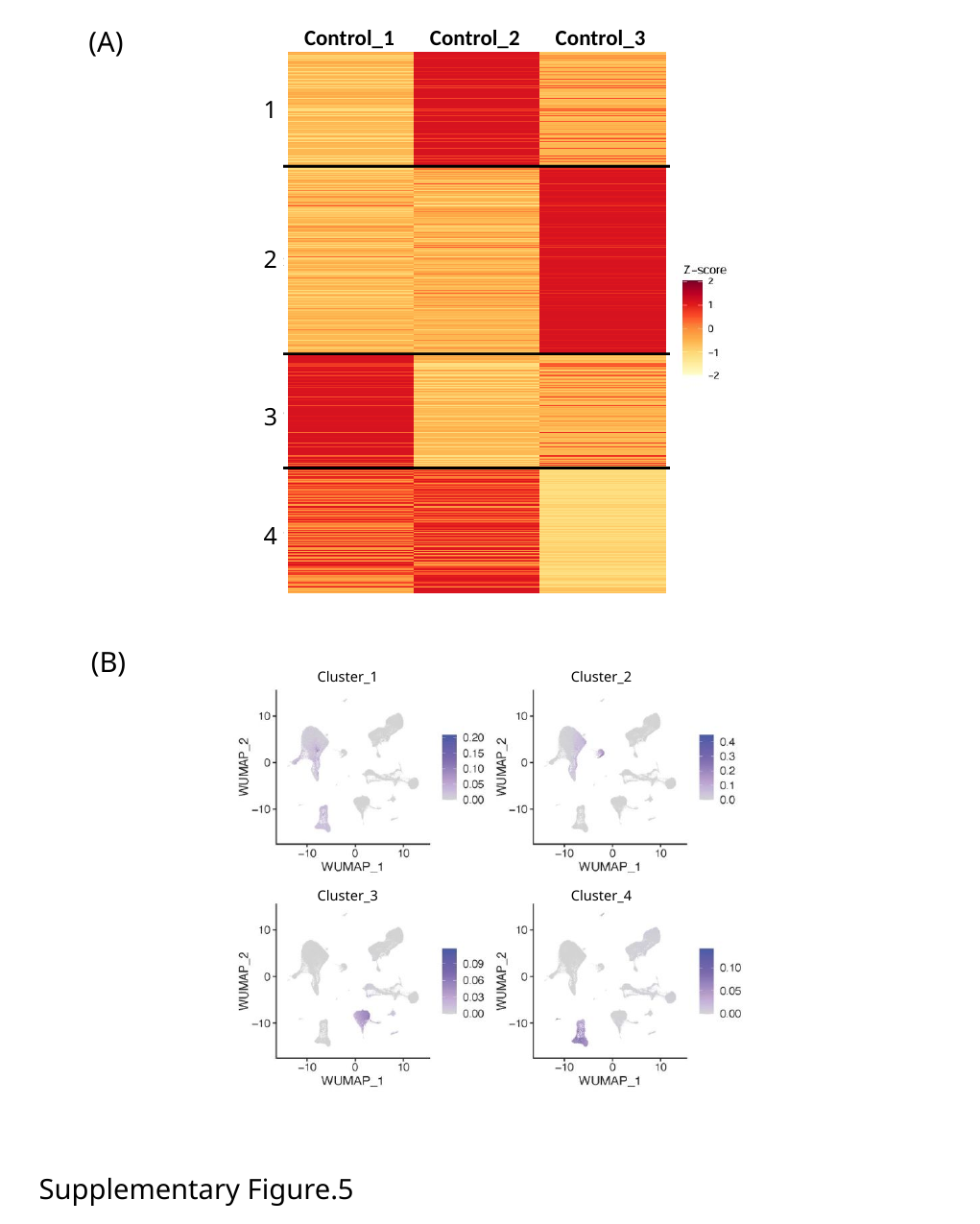

(A)
Control_1
Control_2
Control_3
1
2
3
4
(B)
Cluster_1
Cluster_2
Cluster_3
Cluster_4
Supplementary Figure.5

## Slide 6
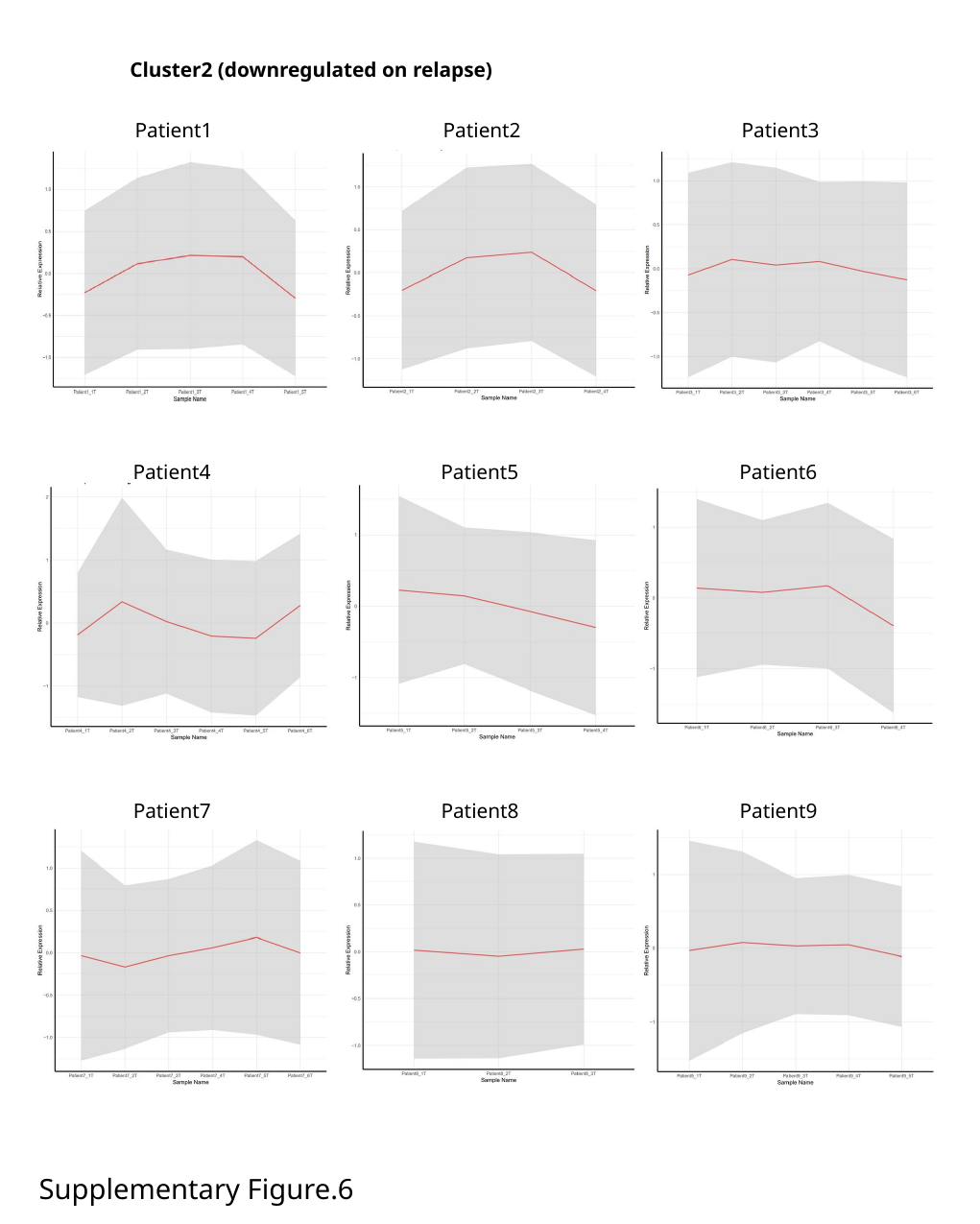

Cluster2 (downregulated on relapse)
Patient1
Patient2
Patient3
Patient4
Patient5
Patient6
Patient7
Patient8
Patient9
Supplementary Figure.6

## Slide 7
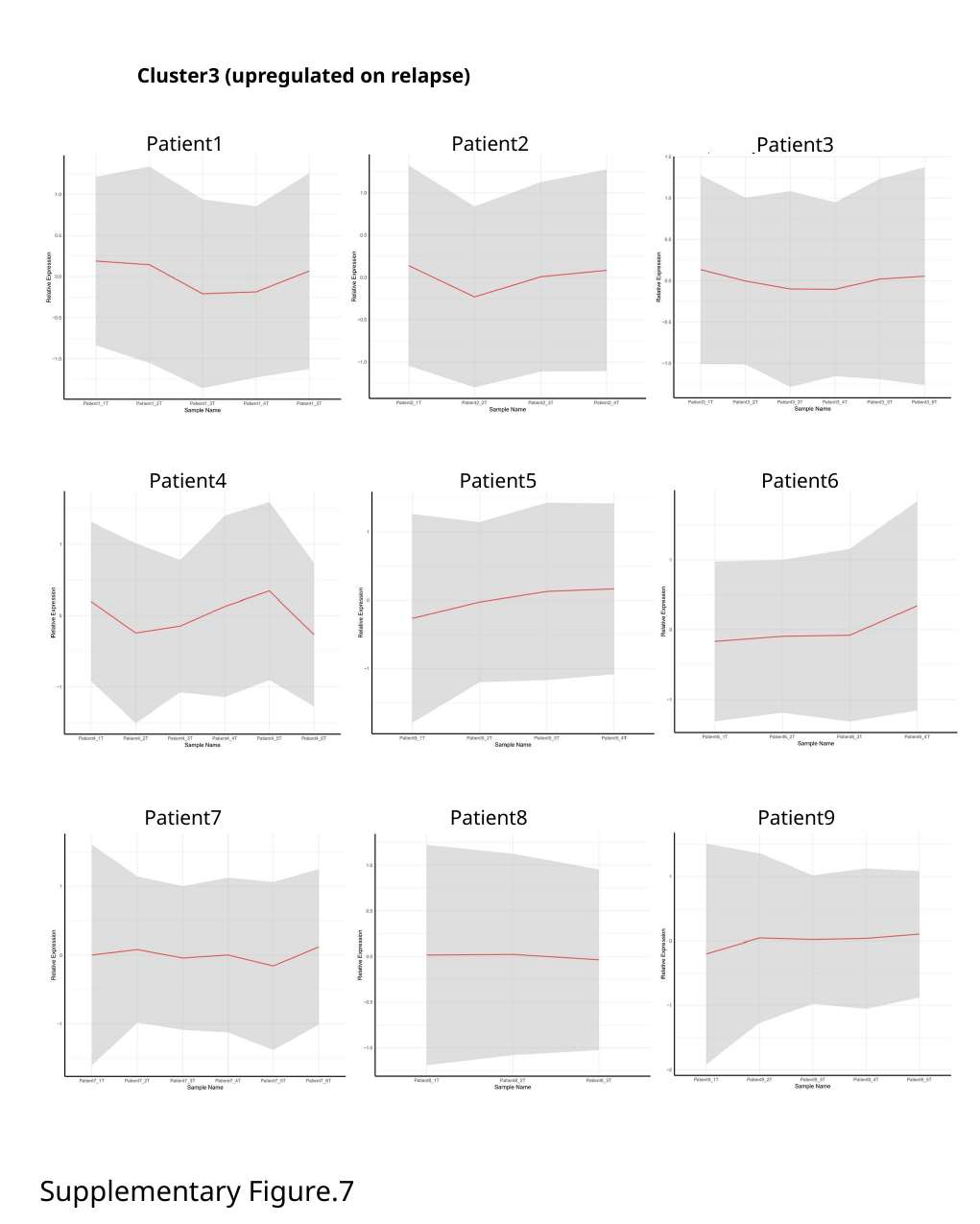

Cluster3 (upregulated on relapse)
Patient1
Patient2
Patient3
Patient6
Patient5
Patient4
Patient8
Patient7
Patient9
Supplementary Figure.7

## Slide 8
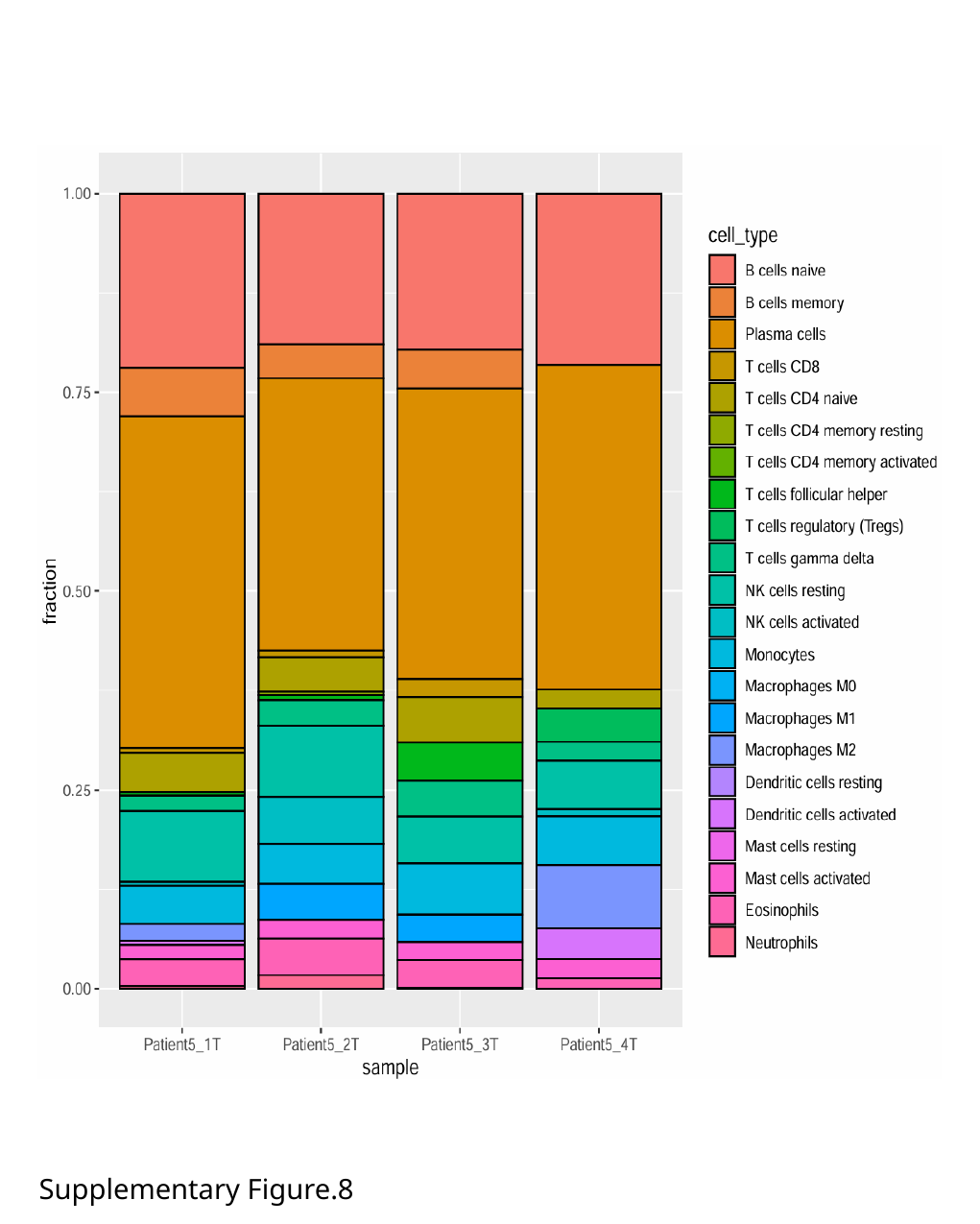

Supplementary Figure.8
